# Sirtuin-1 Regulates Mitochondrial Calcium Uptake Through Mitochondrial Calcium Uptake 1 (MICU1)

**DOI:** 10.3390/life15020174

**Published:** 2025-01-25

**Authors:** Xinyi Zhang, Shuhu Liu, Yanshan Su, Ling Zhang, Ting Guo, Xuemin Wang

**Affiliations:** Key Laboratory of Mental Health of the Ministry of Education, Guangdong-Hong Kong-Macao Greater Bay Area Centre for Brain Science and Brain-Inspired Intelligence, Guangdong-Hong Kong Joint Laboratory for Psychiatric Disorders, Guangdong Provincial Key Laboratory of Psychiatric Disorders, Guangdong Basic Research Center of Excellence for Integrated Traditional and Western Medicine for Qingzhi Diseases, Department of Neurobiology, School of Basic Medical Sciences, Southern Medical University, Guangzhou 510515, China; sunnyzhang246@163.com (X.Z.); lshcraft@163.com (S.L.); 15625044469@163.com (Y.S.); zl_alves@163.com (L.Z.); 13143374395@163.com (T.G.)

**Keywords:** mitochondria, calcium uptake, Sirtuin 1, SIRT1, mitochondrial calcium uptake 1, MICU1

## Abstract

Mitochondria play a central role in cell biological processes, functioning not only as producers of ATP but also as regulators of Ca^2+^ signaling. Mitochondrial calcium uptake occurs primarily through the mitochondrial calcium uniporter channel (mtCU), with the mitochondrial calcium uptake subunits 1, 2, and 3 (MICU1, MICU2, and MICU3) serving as the main regulatory components. Dysregulated mitochondrial calcium uptake is a hallmark of cellular degeneration. Sirtuin 1 (SIRT1), a key regulator of cellular metabolism, plays a critical role in aging and various neurodegenerative conditions. By blocking SIRT1 using EX527 or shSIRT1, we observed mitochondrial structural fragmentation as well as intensified and prolonged mitochondrial calcium overload. Our study revealed a direct interaction between SIRT1 and MICU1. Notably, SIRT1 inhibition resulted in reduced MICU1 expression, hence led to mitochondrial calcium overload, illustrating the unconventional role of SIRT1 in governing mitochondrial function.

## 1. Introduction

Mitochondria serve as the primary energy source within cells, responsible for powering cellular activities. In addition to their energy-generating function, mitochondria also act as a major cytosolic calcium buffering system, playing a critical role in regulating cellular metabolism. However, mitochondrial calcium overload resulting from the imbalance of calcium uptake and efflux leads to enhanced oxidative stress and the generation of reactive oxygen species (ROS), ultimately triggering the activation of apoptotic factors and promoting cell apoptosis [1,2].

The transport of calcium into mitochondria primarily occurs through the mitochondrial calcium uniporter channel (mtCU), comprised of the mitochondrial calcium uniporter (MCU), an inner-membrane protein responsible for channel formation, and the essential MCU regulator (EMRE). The activity of this channel is tightly controlled by the mitochondrial calcium uptake proteins 1, 2, and 3 (MICU1/2/3) [3,4,5]. In particular, MICU1 and MICU2 typically form a heterodimer and jointly regulate MCU activity. Cells maintain calcium uptake from the endoplasmic reticulum to mitochondria by modulating the MCU–MICU1 interaction to ensure a dynamic balance between calcium supply and utilization [6]. Disruption of the MICU1–MICU2 dimer structure or the deletion of MICU1 leads to an increase in mitochondrial calcium concentration, resulting in elevated ROS levels and heightened cellular susceptibility to death [7]. Dysregulation of mitochondrial calcium dynamics has been implicated in various neurodegenerative disorders, including Alzheimer’s disease (AD) and Parkinson’s disease (PD) [8,9,10,11].

Sirtuin1 (SIRT1), a nicotinamide adenine dinucleotide (NAD+)-dependent protein deacetylase, plays essential roles in cellular stress responses, metabolism, and aging. By regulating the acetylation of downstream genes, SIRT1 is involved in inflammatory responses and programmed cell death, thereby influencing the longevity of organisms [12]. In the central nervous system, SIRT1 promotes axon extension, dendritic growth, and synaptic plasticity, thereby influencing memory formation [13,14,15]. Studies have shown that activation of SIRT1 can ameliorate symptoms of neurodegenerative diseases such as AD and PD, and enhance the brain’s resilience to cerebral ischemia-reperfusion injury [16,17,18]. Currently, the regulatory effects of SIRT1 on mitochondria primarily involve the activation of PGC1α-mediated mitochondrial biogenesis, oxidative phosphorylation, and the modulation of nuclear and mitochondrial genes associated with energy production [19,20]. However, while it has been reported that SIRT1 regulates the expression of calcium regulatory genes and its deficiency can lead to disturbances in calcium flux, a thorough understanding of the direct regulatory effects of SIRT1 on mitochondrial function requires additional investigation [21,22,23].

In this study, we investigated the influence of SIRT1 on mitochondrial morphology and calcium uptake functionality in HeLa cells, as well as its downstream regulatory effects on the protein MICU1. Moreover, we sought to validate whether SIRT1 engages in the modulation of mitochondrial calcium uptake through MICU1. EX-527, which inhibits SIRT1 enzymatic activity by occupying the nicotinamide-binding site, was utilized as a pharmacological inhibitor of SIRT1 [24,25].

## 2. Materials and Methods

Studies were conducted in strict accordance with the recommendations outlined in the Guide for the Care and Use of Laboratory Animals of the National Institutes of Health. The protocol was approved by the Committee on the Ethics of Animal Experiments of Southern Medical University (Permit Number: 2016138).

### 2.1. Animals and Cells

SD rats at 17–18 days of gestation were purchased from the Laboratory Animal Center of Southern Medical University. HeLa cells were obtained from the US ATCC cell bank. 

### 2.2. Cell Culture

HeLa cells stored in liquid nitrogen were thawed at 37 °C in a water bath, then transferred aseptically to a 15 mL centrifuge tube within a sterile laminar flow hood. Cells were gently resuspended in 5 mL (DMEM high-glucose base medium + 10% FBS) to recover the osmotic pressure before centrifugation at 900 rpm for 6 min. Following centrifugation, the supernatant was discarded, and 2 mL of the cell pellet was resuspended in medium. The cell suspension (1.0 mL) was added to a 60 mm culture dish, then cells were cultured at 37 °C in a 5% CO_2_ incubator. The culture medium was changed regularly based on cell growth rates, with cells being passaged when reaching 90% confluency using a 0.25% trypsin–0.02% EDTA working solution. To restrain the expression of SIRT1, EX527 (Sigma, St. Louis, MO, USA), an inhibitor of SIRT1, was dissolved in dimethyl-sulfoxide (DMSO, Sigma, St. Louis, MO, USA) and added to the cell culture to achieve a final concentration of 10 μM [26]. The control group received the same amount of DMSO.

Surgical instruments were sterilized in 75% alcohol, flame sterilized, and exposed to ultraviolet light. Pregnant SD rats at 17–18 days of gestation underwent aseptic surgery under anesthesia euthanasia with 10% pentobarbital sodium. Fetal mice were decapitated, and brain tissues were harvested. After removing blood vessels and meninges, the cerebral cortex was dissected, cut into 1 mm^3^ fragments, and digested with 0.25% trypsin at 37 °C. Following digestion, a single-cell suspension was prepared for culturing cortical neurons in a 5% CO_2_ incubator at 37 °C. Neurons were identified and utilized for experiments after 7 days of culture.

### 2.3. Mitochondrial Extraction

Mitochondrial extraction was performed using the KEYGEN Biologics mitochondrial extraction kit (KGA3106, Nanjing, China). Briefly, HeLa cells were lysed using Buffer and subjected to centrifugation to obtain the mitochondrial fraction. The obtained mitochondria were washed and stored at −80 °C for subsequent experiments.

### 2.4. Western Blot Analysis

HeLa cells were digested and lysed, and total cell proteins were extracted and quantified using a BCA protein quantification kit (Thermo Fisher Scientific, Eugene, OR, USA). The samples were loaded onto SDS-PAGE gels for electrophoresis, followed by transfer to PVDF membranes. Membranes were blocked and then probed with diluted primary antibodies. After overnight incubation at 4 °C, membranes were washed and incubated with secondary antibodies. The protein bands were visualized using chemiluminescent reagents. Anti-SIRT1 (1:1000, H-300: sc-15404, Santa Cruz Biotechnology, Santa Cruz, CA, USA), anti-LaminB (1:1000, BA1228, Boster, Wuhan, China), anti-α-tubulin (1:5000, T9026, Sigma, USA) and anti-COXIV (1:1000, 11242-1-AP, ProteinTech, Manchester, UK) were use to detect the expression of SIRT1 in mitochondria of HeLa cells. To detect the protein levels of MCU and MICU1 proteins in HeLa cells, anti-MCU (1:1000, 26312-1-AP, ProteinTech, Manchester, UK) and anti-MICU1 (1:1000, #12524, Cell Signaling, Danvers, MA, USA) were used.

### 2.5. Cell Immunofluorescence

After washing once in 1× Phosphate Buffered Saline (PBS), the experimental cells were fixed using a 4% paraformaldehyde solution (in 1× PBS) at room temperature for 15 min. Subsequently, the cells were permeabilized, blocked with a 0.3% Triton X-100 and 5% Bovine Serum Albumin (BSA) blocking solution (in 1× PBS) at room temperature for 30 min, and incubated with primary antibodies overnight. To detect SIRT1 in cells, anti-SIRT1 (1:200, 13161-1-AP, Proteintech, Wuhan, China) was employed. Following overnight incubation, the cells were washed in 1× PBS three times at room temperature and stained with secondary antibodies (1:200) at room temperature for 1 h. After washing, the cells were examined under a confocal fluorescence microscope. Cell experiments pre-treated with MitoTracker (100 nM for 20 min, Thermo Fisher Scientific, Eugene, OR, USA), a red fluorescent dye specifically targeting mitochondria, required complete darkness throughout the entire process. Images were taken using a Nikon Ti microscope with a 60 × 1.4 NA oil immersion objective, at Zoom 1, using a Nikon A1plus camera and controlled by NIS-Elements 5.3. Signals were captured sequentially into one or two channel modes. Manders’ colocalization coefficients (MCCs) were employed to quantify the degree of colocalization between SIRT1 and mitochondria, as well as between SIRT1 and MICU1. Background correction was performed using selected regions of interest (ROIs). An MCC value greater than 0.6 indicated significant colocalization [27]. To assess mitochondrial fragmentation, we utilized the Mitochondrial Network Analysis (MiNA) toolset in ImageJ 2.0.0 (National Institutes of Health, Stapleton, NY, USA), measuring the area occupied by mitochondrial structures (mitochondrial footprint) and the mean length of branches (mean branch length) [28].

### 2.6. RNA Interference

shNC, shSIRT1, and shSIRT2 were purchased from Gima Biology (Suzhou, China).

shSIRT1: 5′-GAAGTGCCTCAGATATTAA-3′,

shSIRT2: 5′-GACTCCAAGAAGGCCTACA-3′.

The interfering plasmid vector contained a GFP fluorescent reporter gene to evaluate transfection efficiency. When the cell density reached 70–80%, HeLa cells were transfected with shNC, shSIRT1, or shSIRT2 using Lipofectamine 2000 (Thermo Fisher Scientific, Eugene, OR, USA) in serum-free medium according to the manufacturer’s instructions.

### 2.7. Bacterial Plasmid Transformation

A total of 50 ng each of pCMV-HA, MICU1-HA, PCDNA3-CFP, and SIRT1-CFP was added into 50 μL of prepared Top10 competent cells and placed on ice for 30 min, then transferred to a metal bath at 42 °C for 90 s, and rapidly returned to ice for 2 min. Subsequently, 200 μL of antibiotic-free LB liquid medium was added to the bacterial solution and incubated at 37 °C with shaking for 1 h. The cultures were evenly spread on solid LB agar plates containing ampicillin and incubated at 37 °C for 12 to 16 h until single colonies were large enough to be selected for expansion culture. Plasmids were extracted using the Kangwei Century Endotoxin-Free Extraction Kit (Taizhou, China) following sequencing.

### 2.8. Cell Transfection

HeLa cells were transfected with liposomes followed by Lipofectamine 2000 manual. Before transfection, the culture medium was substituted with the DMEM high-glucose base medium without FBS or antibiotics. The plasmids and Lipofectamine 2000 (Thermo Fisher Scientific, Eugene, OR, USA) for transfection were diluted in Opti-MEM medium respectively and were mixed at a ratio of 1:1. After 5 min incubation at room temperature, the DNA–lipid complex was added into cells and incubated at room temperature for an additional 6 h. After this time, DMEM was replaced with complete medium for further culture. Cellular status was assessed 24 h post-transfection.

### 2.9. Glutathione S-Transferase (GST) Pull-Down Assay

The GST-SIRT1 and GST control proteins were induced and purified in Escherichia coli, then absorbed on the corresponding agarose beads. A single bacterial colony containing either the pGEX-4T-3 or pGEX-4T-3-SIRT1 plasmid was amplified overnight. On the second day, each bacterial solution was diluted into 50 mL of sterile LB liquid medium at a ratio of 1:100, and ampicillin was added to a final concentration of 50 μg/mL. Incubation continued at 37 °C for 4 h, and isopropyl-β-D-thiogalactopyranoside (IPTG) with a final concentration of 0.5 mM was added to the medium to induce the expression of GST-SIRT1 or GST control proteins. Then, 6–8 h later, the bacterial cultures were lysed ultrasonically on ice in PBS containing protease inhibitors. The resulting supernatants contained either GST-SIRT1 or GST control proteins. Glutathione Sepharose 4B (GE Healthcare, Camarillo, CA, USA) was added to the above solution, and the agarose microbeads combining with GST-SIRT1 or GST control proteins were obtained after mixing at room temperature. HeLa cells were transfected with MICU1-HA plasmid, and the cells were collected 24 h after transfection. After ultrasonic lysis, the cell lysates were mixed with the agarose beads. Bound proteins were analyzed via immunoblotting. Anti-HA (1:1000, 05-902R, Sigma, St. Louis, MO, USA) and anti-GST (1:1000, SAB2702378, Sigma, St. Louis, MO, USA) were used. 

### 2.10. Co-Immunoprecipitation (Co-IP)

Protein A agarose beads (sc-2001, Santa Cruz Biotechnology, Santa Cruz, CA, USA) were added into the lysed cells and rotated at 4 °C for 1 h to bind non-specific proteins. In total, 2 μg of Flag or SIRT1 antibody was added to the cell lysate supernatant and rotated at 4 °C overnight. Protein A agarose beads were added to samples and rotated at 4 °C for 1 h. After boiling, the samples were detected by Western blot. Anti-Flag (1:1000, F1804, Sigma, St. Louis, MO, USA), anti-HA (1:1000, SAB4300603, Sigma, St. Louis, MO, USA), and anti-SIRT1 (1:1000, H-300: sc-15404, Santa Cruz Biotechnology, Santa Cruz, CA, USA) were used.

### 2.11. Mitochondrial Calcium Signal Detection

HeLa cells were incubated with 1 µM of rhodamine-2-acetoxymethyl ester (Rhod-2 AM; Thermo Fisher Scientific, Eugene, OR, USA) or transfected with mitochondrial calcium-targeting fluorescence plasmid CMV-mito-R-GECO1 to assess mitochondrial calcium levels. Changes in fluorescence signals were monitored using confocal microscopy after treatment with mitochondrial calcium influx inducers. Fluorescence observations were recorded and analyzed for calcium signal dynamics. Images were acquired as single frames every 4 s for a total duration of 300 s using a Nikon Ti microscope equipped with a 40 × 1.4 NA oil immersion objective at 1× zoom. The Nikon A1 Plus camera was used, controlled by NIS-Elements 5.3 (Nikon, Tokyo, Japan). Signals were captured sequentially in a single-channel mode. To induce calcium release from the endoplasmic reticulum into the cytoplasm, 100 μM histamine (Sigma, St. Louis, MO, USA) was applied at the 60 s time point.

### 2.12. Quantification and Statistical Analysis

All experiments were performed in 3 independent replicates, and all values were presented as mean ± standard deviation (SD). GraphPad Prism 9.0 software (San Diego, CA, USA) was used. The two groups were compared with Student’s *t*-test. The relative protein level in the Western blot were compared with the Mann–Whitney U Test. Data sets that involved more than two groups were assessed by one-way ANOVA, followed by least-significant difference (LSD) post hoc tests. Comparisons among four groups based on two variables were performed with two-way ANOVA, followed by Tukey’s multiple comparison post-tests for post hoc analysis. All statistical tests were two-sided, and *p* < 0.05 was considered statistically significant. Significance levels * *p* < 0.05; ** *p* < 0.01; *** *p* < 0.001; **** *p* < 0.0001.

## 3. Results

### 3.1. Long-Term Inhibition of SIRT1 Alters Mitochondrial Morphology in HeLa Cells

**Figure 1:**
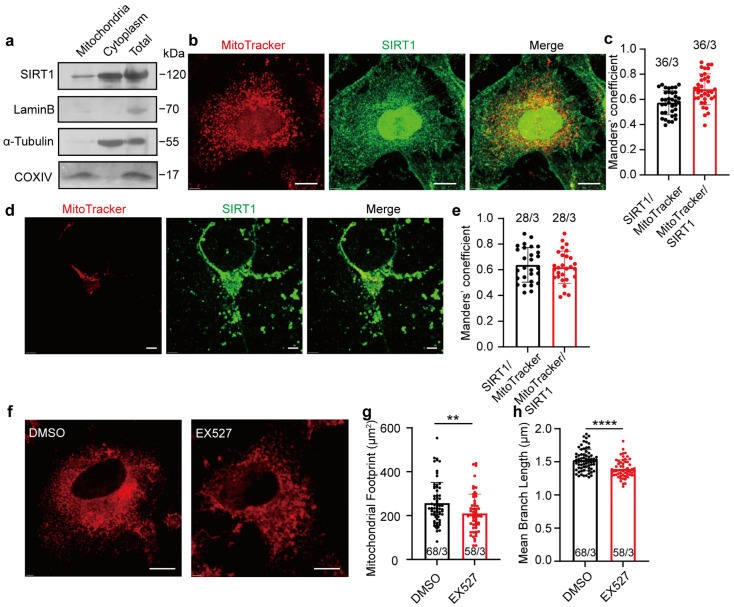
Expression of SIRT1 in mitochondria of HeLa cells and rat cortical neurons. (**a**) Western blot analysis of SIRT1 expression in mitochondria, cytoplasm, and whole-cell lysates of HeLa cells. (**b**) Confocal microscopy determining the SIRT1 colocalization with mitochondria in HeLa cells. Red, MitoTracker; Green, SIRT1. Magnification is 60×. Scale bars, 5 μm. (**c**) The levels of colocalization between SIRT1 and mitochondria expressed as MCC for a fraction of SIRT1 pixels that are shared with mitochondria (black bar) or vice versa (red bar) in HeLa cells. (**d**) Confocal microscopy determining the SIRT1 colocalization with mitochondria in SD rat cortical neurons. Red, MitoTracker; Green, SIRT1; Blue, Dapi. Magnification is 60×. Scale bars, 5 μm. (**e**) The levels of colocalization between SIRT1 and mitochondria expressed as MCC for a fraction of SIRT1 pixels that are shared with mitochondria (black bar) or vice versa (red bar) in SD rat cortical neurons. (**f**) Representative images of confocal microscopy determining 10 μM of EX527 to mitochondrial morphology in HeLa cells. Red, MitoTracker. Magnification is 60×. Scale bars, 5 μm. (**g**,**h**) The footprint of mitochondria and mean branch length of HeLa cells treated with DMSO or EX527 for 1 h. Data presented as mean ± standard deviation (SD). ** *p* < 0.01, **** *p* < 0.0001. The number of cells analyzed/the number of independent experiments is indicated in the bars.

The expression of SIRT1 in mitochondria was initially examined. Western blot analysis confirmed the presence of SIRT1 in isolated mitochondria derived from HeLa cells (Figure 1a; full blots are shown in Supplementary Figure S1a). To evaluate colocalization between SIRT1 and mitochondria, anti-SIRT1 antibodies and MitoTracker were employed to visualize respective expression in HeLa cells, and Manders’ colocalization coefficients (MCCs) were used to quantify the colocalization. We observed a substantial proportion of MitoTracker signals demonstrating colocalization with SIRT1 immunosignals (MCC = 0.68 ± 0.1226, *n* = 36; Figure 1b,c, red bar). A smaller fraction of SIRT1 signals colocalized with MitoTracker signals (MCC = 0.57 ± 0.0959, *n* = 36; Figure 1b,c, black bar), likely attributable to the broader spatial distribution of SIRT1 within cellular environments (additional images are provided in Supplementary Figure S1b). Primary cultured SD fetal rat cortical neurons exhibited mitochondrial-SIRT1 colocalization patterns (SIRT1 to MitoTracker: MCC = 0.64 ± 0.1344, *n* = 28; Figure 1d,e, black bar. MitoTracker to SIRT1: MCC = 0.62 ± 0.1258, *n* = 28; Figure 1d,e, red bar. Additional images are provided in Supplementary Figure S1c). Furthermore, the inhibition of SIRT1 in HeLa cells using the specific inhibitor EX527 for 1 h led to pronounced mitochondrial fragmentation, with a decreased mitochondrial footprint and diminished mean branch length (Figure 1f–h; additional images are provided in Supplementary Figure S1d). These findings emphasize the localization of SIRT1 within mitochondria and its crucial role in maintaining mitochondrial morphology.

### 3.2. SIRT1 Inhibitor EX527 Enhanced Histamine-Induced Mitochondrial Calcium Overload in HeLa Cells

**Figure 2:**
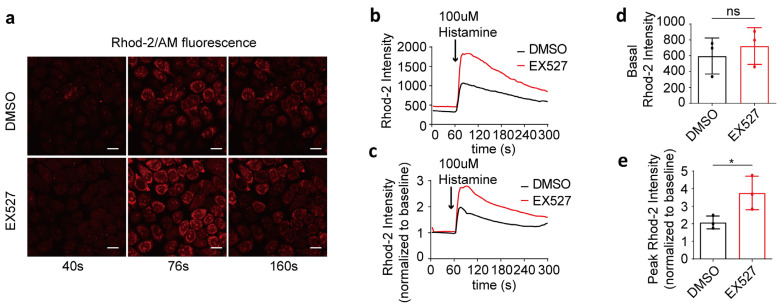
EX527 affected mitochondrial calcium uptake in HeLa cells. (**a**) Representative confocal microscopy images acquired at 40 s, 76 s, and 160 s depict mitochondrial calcium levels in HeLa cells following treatment with either DMSO or 10 μM EX527 for 1 h. Continuous recording was performed, with one frame captured every 4 s. At the 60 s time point, 100 μM histamine was added. Rhod-2 AM; Magnification is 40×. Scale bars, 20 μm. (**b**) The mitochondrial calcium level in HeLa cells treated with DMSO or EX527 for 1 h was determined by Rhod-2 AM staining. Overall, 100 μM histamine was used to induce cellular calcium release. (**c**) The fold change in mitochondrial calcium level in HeLa cells treated with DMSO or EX527 for 1 h, normalized to baseline. (**d**) Average Rhod-2 AM fluorescence intensity before 100 μM histamine treatment. (**e**) Maximum fold change in Rhod-2/AM fluorescence intensity after 100 μM histamine treatment. For each group, 30 cells from three independent experiments were analyzed. The data are presented as mean values ± SD. ns, not significant; * *p* < 0.05.

To investigate the impact of SIRT1 on mitochondrial calcium uptake, 100 μM of histamine was utilized to induce the release of calcium from the endoplasmic reticulum into the cytoplasm. The mitochondrial calcium levels were quantified by measuring the intensity of Rhod-2/AM fluorescence (Figure 2a–c). Prior to histamine stimulation, no significant differences in mitochondrial calcium concentrations were observed between the two groups (Figure 2d). Subsequent to histamine administration, the maximum fold change in mitochondrial calcium concentration in EX527-treated cells was markedly higher than that in the DMSO control group, indicating a mitochondrial calcium overload in EX527-treated HeLa cells (Figure 2e).

### 3.3. SIRT1 shRNA Exacerbates Histamine-Induced Mitochondrial Calcium Overload in HeLa Cells

**Figure 3:**
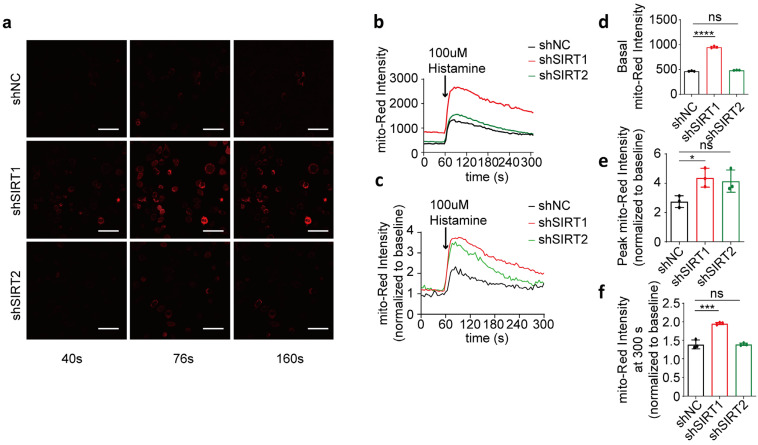
shSIRT1 affected mitochondrial calcium uptake in HeLa cells. (**a**) Representative confocal images (40 s, 76 s, and 160 s) of mitochondrial calcium in HeLa cells co-transfected with shSIRT1 or shSIRT2 interference plasmids and the CMV-mito-R-GECO1 mitochondrial calcium-targeting fluorescence plasmid, followed by treatment with 100 μM histamine to induce cellular calcium release. Continuous recording was performed, with one frame captured every 4 s. At the 60 s time point, 100 μM histamine was added. Red, mito-Red; Magnification is 40×. Scale bars, 50 μm. (**b**) Mitochondrial calcium fluorescence intensity changes in HeLa cells determined by mito-Red intensity. Overall, 100 μM histamine was used to induce cellular calcium release. (**c**) The fold change in mitochondrial calcium level in HeLa cells normalized to baseline. (**d**) Average mitochondrial calcium fluorescence intensity before treatment with 100 μM histamine. (**e**) Maximum fold change in mitochondrial calcium fluorescence intensity after treatment with 100 μM histamine. (**f**) The fold change in mitochondrial calcium fluorescence intensity at 300 s. For each group, 30 cells from three independent experiments were analyzed. The data are presented as mean values ± SD. ns, not significant; * *p* < 0.05; *** *p* < 0.001; **** *p* < 0.0001.

To assess the specific effect of SIRT1 on mitochondrial calcium uptake, we transiently suppressed the expression of SIRT1 or SIRT2 in HeLa cells using shRNA interference (Figure 3a–c; full blots are shown in Supplementary Figure S2b). Our findings revealed that the basal mitochondrial calcium concentration in the shSIRT1 group was significantly higher than in the shNC group 48 h after co-transfection, while the basal intensity of the shSIRT2 group did not show a significant difference compared to the shNC group (Figure 3d). Following histamine-induced calcium release from the endoplasmic reticulum, the increase in the mitochondrial calcium level in the shSIRT1 group was consistently higher than in the shNC group, characterized by a significantly higher rate of increase in mito-Red fluorescence intensity. In contrast, the shSIRT2 group did not show a significant increase (Figure 3e). Furthermore, the rate of reduction in mitochondrial calcium levels in the shSIRT1 group was slower compared to that in the shNC group, with a significantly higher fold change observed in mitochondrial calcium levels at 240 s post-histamine administration, whereas no significant differences were observed in the intensity within the shSIRT2 group relative to the shNC group (Figure 3f). This suggests that disruption of SIRT1 impairs mitochondrial regulation of basal calcium levels and enhances histamine-triggered calcium uptake. In contrast, SIRT2 interference does not elicit this response.

### 3.4. MICU1 and SIRT1 Interacted with Each Other

**Figure 4:**
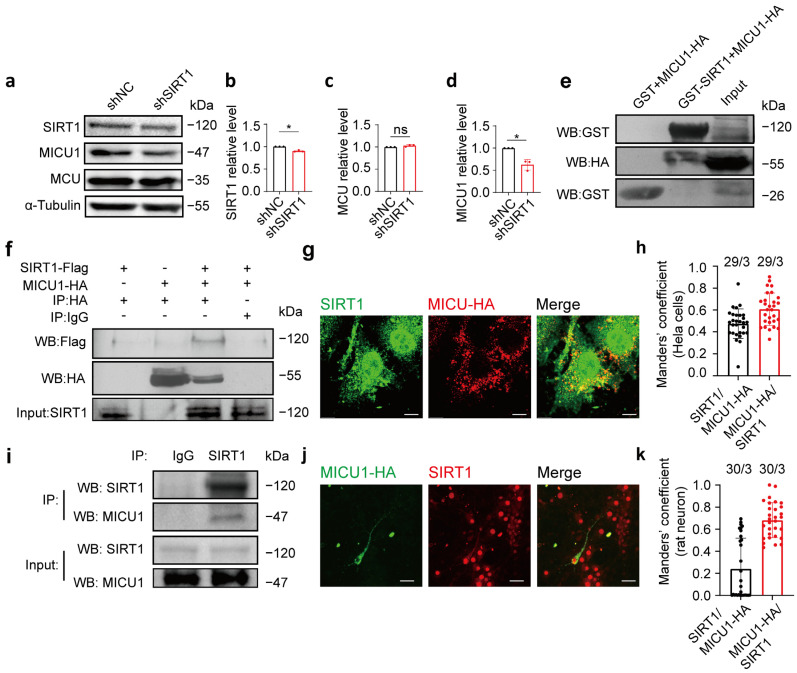
MICU1 and SIRT1 interacted with each other. (**a**–**d**) SIRT1, MCU, and MICU1 protein expression levels after HeLa cells transfection of shSIRT1 (*n* = 3 biological replicates; three technical replicates were achieved). (**e**) GST assay was performed to determine the interaction of SIRT1 and MICU1. Purified GST and GST-SIRT1 proteins were incubated with cell lysate (Input) containing MICU1-HA proteins, followed by examination of the proteins bound to GST and GST-SIRT1 via Western blot analysis. (**f**) Co-IP was determined in SIRT1 immunoprecipitates by Western blot analysis. SIRT1-flag + pcmv-HA, pcmv-tag4a + MICU1-HA, SIRT1-flag + MICU1-HA, and SIRT1-flag + MICU1-HA constructs were expressed in HeLa cells, and anti-FLAG, anti-HA, and anti-SIRT1 antibodies were employed for immunoprecipitation. (**g**) SIRT1 and MICU1 exhibited colocalization in HeLa cells. Red, HA; Green, SIRT1. Magnification is 60×. Scale bars, 5 μm. (**h**) The levels of colocalization between SIRT1 and MICU1-HA expressed as MCC for a fraction of SIRT1 pixels that are shared with HA (black bar) or vice versa (red bar) in HeLa cells. (**i**) Lysates from rat neurons cultured for 7 days were collected. SIRT1 antibody was utilized for immunoprecipitation, and anti-SIRT1 and anti-MICU1 antibodies were used for Western blot analysis. (**j**) MICU1-HA was transfected into primary cultured rat neurons, and antibodies targeting HA and SIRT1 were applied as immunofluorescent co-labels. Red, SIRT1; Green, HA. Magnification is 60×. Scale bars, 50 μm. (**k**) The levels of colocalization between SIRT1 and mitochondria expressed as MCC for a fraction of SIRT1 pixels that are shared with HA (black bar) or vice versa (red bar) in SD rat cortical neurons. The data are presented as mean values ± SD. ns, not significant; * *p* < 0.05. Statistical analysis was carried out using the Mann–Whitney U Test (**b**–**d**). The number of cells analyzed/the number of independent experiments is indicated above the bars.

We subsequently examined whether SIRT1 modulates the protein level of MICU1. SIRT1 expression was knocked down using shSIRT1 in HeLa cells, and the protein levels of MICU1 and MCU were measured 48 h following transfection. The results indicated that after 48 h of transfection, the MICU1 protein level decreased in the shSIRT1 group, while the MCU protein level remained unaltered (Figure 4a–d; full blots are shown in Supplementary Figure S2a).

To investigate the interaction between SIRT1 and MICU1, we examined their binding both intracellularly and extracellularly. First, we overexpressed the MICU1-HA plasmid in HeLa cells, extracted cell lysates after lysis, and conducted a GST pull-down assay with GST and GST-SIRT1 expressed in bacteria. Following purification, GST-SIRT1 or GST was incubated with the cell lysate containing the MICU1-HA plasmid. The results demonstrated that GST-SIRT1 was bound to MICU1-HA, indicating an interaction between SIRT1 and MICU1 (Figure 4e; full blots are shown in Supplementary Figure S2b). Next, HeLa cells were transfected with SIRT1-flag plus pCMV-HA, pCMV-tag4a plus MICU1-HA, or SIRT1-flag plus MICU1-HA, and lysed after 24 h for co-immunoprecipitation. The results indicated an intracellular interaction between SIRT1 and MICU1 (Figure 4f; full blots are shown in Supplementary Figure S2c). Fluorescence localization analysis of SIRT1 and MICU1 indicated their colocalization in HeLa cells. A large proportion of HA signals colocalized with SIRT1 signals (MCC = 0.61 ± 0.1482, *n* = 29; Figure 4g,h, red bar). A smaller fraction of SIRT1 signals were found colocalized with HA signal (MCC = 0.47 ± 0.1349, *n* = 29; Figure 4g,h, black bar. Additional images are provided in Supplementary Figure S2d). To confirm the endogenous interaction between SIRT1 and MICU1, we performed co-immunoprecipitation experiments using primary cultures of rat cortical neurons. These results further confirmed the interaction between SIRT1 and MICU1 in primary cultured rat neurons (Figure 4i; full blots are provided in Supplementary Figure S2e). Fluorescence localization analysis of SIRT1 and MICU1 indicated their colocalization in primary cultured rat cortical neurons. A substantial fraction of HA signals colocalized with SIRT1 signals (MCC = 0.68 ± 0.1589, *n* = 30; Figure 4j,k, red bar). A smaller fraction of SIRT1 signals exhibited colocalization with MICU1-HA signals (MCC = 0.24 ± 0.2776, *n* = 30; Figure 4j,k, black bar. More images of transfected neurons are shown in the Supplementary Material Figure S2d. Additional images are provided in Supplementary Figure S2f). These findings collectively indicate an interaction between SIRT1 and MICU1, suggesting that MICU1 could play a role in SIRT1-mediated regulation of mitochondrial calcium homeostasis.

### 3.5. Overexpression of MICU1 Mitigated the Mitochondrial Calcium Overload Induced by SIRT1 Inhibition

**Figure 5:**
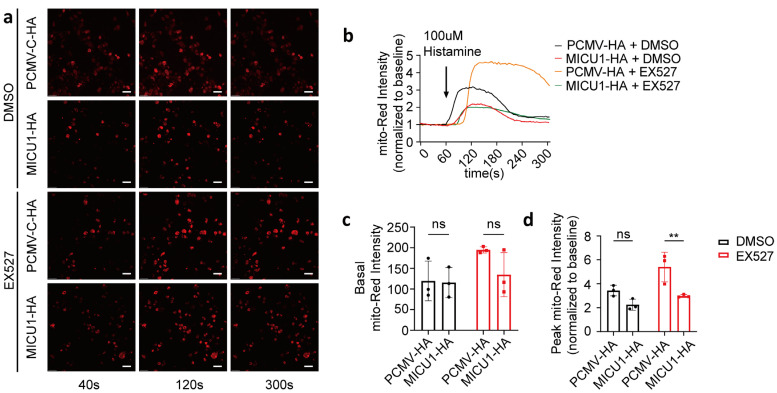
SIRT1 regulated mitochondrial calcium uptake through MICU1. (**a**) HeLa cells were co-transfected with MICU1-HA or pCMV-C-HA, along with the mitochondrial calcium-targeting fluorescent plasmid CMV-mito-R-GECO1. Representative confocal images (40 s, 120 s, and 300 s) of mitochondrial calcium in HeLa cells treated with DMSO or 10 μM EX527 for 1 h. Overall, 100 μM Histamine was used to induce cellular calcium release. Red, mito-Red; Magnification is 40×. Scale bars, 50 μm. Continuous recording was performed, with one frame captured every 4 s. At the 60 s time point, 100 μM histamine was added. (**b**) The fold change in mitochondrial calcium levels in HeLa cells, normalized to baseline values. (**c**) Average mitochondrial calcium fluorescence intensity before treatment with 100 μM histamine. (**d**) The fold change in the peak mitochondrial calcium fluorescence intensity following treatment with 100 μM histamine. For each group, 30 cells from three independent experiments were analyzed (two-way ANOVA and Tukey’s post hoc test). The data are presented as mean values ± SD. ns, not significant; ** *p* < 0.01.

To investigate whether SIRT1 regulates mitochondrial calcium uptake via MICU1, we overexpressed MICU1 and assessed mitochondrial calcium uptake in the presence of EX527 (Figure 5a,b). Our results demonstrated that 1 h after EX527 treatment, the activation levels of mitochondrial calcium in HeLa cells overexpressing MICU1, following histamine-induced calcium release, were significantly lower compared to those transfected with the control vector. Conversely, no significant differences were detected in basal intensity or activation levels under DMSO conditions (Figure 5c,d). These findings suggest that MICU1 may serve as a regulatory factor in SIRT1-mediated mitochondrial calcium uptake.

## 4. Discussion

Our study elucidated the role of SIRT1 in mitochondrial calcium uptake, highlighting that SIRT1 directly interacts with MICU1 to maintain mitochondrial integrity and Ca^2+^ homeostasis. The loss of SIRT1 function resulted in decreased MICU1 expression, which subsequently led to mitochondrial morphological fragmentation and reduced efficiency in regulating calcium uptake. Abnormal mitochondrial calcium metabolism may contribute to irregular cell discharge and cell death.

Mitochondrial dysfunction is considered one of the primary mechanisms underlying degenerative diseases. While SIRT3 to SIRT5 are known to be located in the mitochondrial matrix [29], only SIRT3 has been associated with neurodegenerative diseases through its regulation of calcium homeostasis [30,31]. SIRT1 has been found to play a key role in maintaining mitochondrial function, promoting mitochondrial biogenesis, and regulating the autophagy–lysosome pathway [32,33]. Previous studies have shown that SIRT1 maintains mitochondrial homeostasis through a variety of substrates. SIRT1 overexpression restores the structure and function of mitochondria by activating Sirtuin 3 (SIRT3) through increasing the deacetylation of SIRT3 [34]. SIRT1 activity decreases the lysine acetylation status in tuberous sclerosis complex 2 (TSC2), regulating mTORC1 signaling and the maintenance of the mitochondrial quality control [35]. Enhancement of the enzymatic activity of SIRT1 leads to increased expression of peroxisome proliferator-activated receptor-gamma coactivator-1alpha (PGC1α) and mitochondrial dynamics protein 18 (MTP18); consequently, this promotes mitochondrial biogenesis and fission, thereby preserving the mitochondrial population in oral cancer cells [36]. These findings together underscore SIRT1′s critical role in maintenance of the stability of mitochondrial structure and function. We found that SIRT1 deficiency induced mitochondria fragmentation and dysfunction in calcium uptake, which could be ameliorated by overexpression of MICU1. The results together suggest a protective role of SIRT1 in maintaining mitochondrial morphology and the function of calcium uptake through MICU1, which further proves the importance of SIRT1 in regulating mitochondrial function.

Our results indicate that SIRT1 interacts with MICU1 to regulate calcium uptake. MICU1, an MCU-mediated mitochondrial calcium uptake protein, acts as a crucial gatekeeper of cellular survival [37]. MICU1 defects can lead to alterations in mitochondrial morphology and calcium uptake kinetics [38,39]. In mice, silencing MICU1 expression in the heart, skeletal muscle, or liver leads to mCa^2+^ overload and impaired uptake, making these organs more susceptible to injury or regeneration impairment, thereby establishing MICU1’s indispensable role in calcium uptake [37,40,41]. Additionally, MCU and MICU1 expression levels are negatively correlated with age, potentially explaining the increased incidence of neurodegenerative diseases with aging [42]. Studies have demonstrated that SIRT1 inhibition could induce acetylation of MCU residue K332, leading to calcium overload and cell depolarization, culminating in cell death [43]. In our experimental work utilizing HeLa cells, interfering with SIRT1 expression did not alter MCU protein levels but significantly diminished MICU1 protein levels, which suggests that SIRT1 plays a crucial role in the maintenance of MICU1. Moreover, overexpression of MICU1 rescued the calcium overload caused by SIRT1 loss, suggesting that SIRT1 maintains mitochondrial calcium metabolism stability through MICU1. This underscores MICU1’s pivotal role in the calcium uptake process [37].

Our findings on the interaction between SIRT1 and MICU1 raise the question of whether SIRT1 exerts its protective effects in various neurodegenerative diseases through this pathway. Given that impairments in Ca^2+^ homeostasis have been documented in patients with neurodegenerative diseases such as AD [44], PD [45], and Huntington’s disease (HD) [46], and considering that neurons from MICU1 knockout (KO) mice and MICU1-deficient patient-derived cells exhibit increased susceptibility to mitochondrial Ca^2+^ overload, excitotoxicity, and cell death [47], it is warranted to further investigate whether the SIRT1-regulated pathway of mitochondrial calcium uptake via MICU1 plays a role in neuronal secondary injury or death in these conditions. This study opens new avenues for exploring the neuroprotective mechanisms of SIRT1.

## Data Availability

The data are available from the corresponding author (xmwang@smu.edu.cn) on reasonable request.

## Supplementary Materials

The following supporting information can be downloaded at: https://www.mdpi.com/article/10.3390/life15020174/s1, Supplementary Figure S1. a. Full blots of Western blot analysis of SIRT1 expression in mitochondria, cytoplasm and whole-cell lysates of Hela cells. b. Additional images of the colocalization of SIRT1 and MitoTracker in Hela cells. c. Additional images showing the colocalization of SIRT1 and MitoTracker in rat cortical neurons. d. Additional images showing the morphology of Hela cells treated with EX527. Supplementary Figure S2. a. Full blots of Western blot analysis of SIRT1, MCU and MICU1 protein expression levels. b. Full blots of Western blot analysis of the GST pull down assay. c. Full blots of Western blot analysis of the co-IP assay in HeLa cells. d. Additional images of the colocalization of SIRT1 and MICU1-HA. e. Full blots of Western blot analysis of the co-IP assay in primary cultured rat neurons. f. Supplementary images of primary cultured rat neurons transfected with MICU1-HA. Red, SIRT1; Green, HA. Magnification is 60×. Scale bars, 50 μm.
