# Supplementary material for: Sirtuin-1 Regulates Mitochondrial Calcium Uptake Through Mitochondrial Calcium Uptake 1 (MICU1)
Source: Life (Basel). 2025 Jan 25;15(2):174. doi: 10.3390/life15020174 (PMC11856031; doi:10.3390/life15020174)
Supplement: Supplementary file 1 [file life-15-00174-s001.zip › life-3434908-supplementary.pdf]

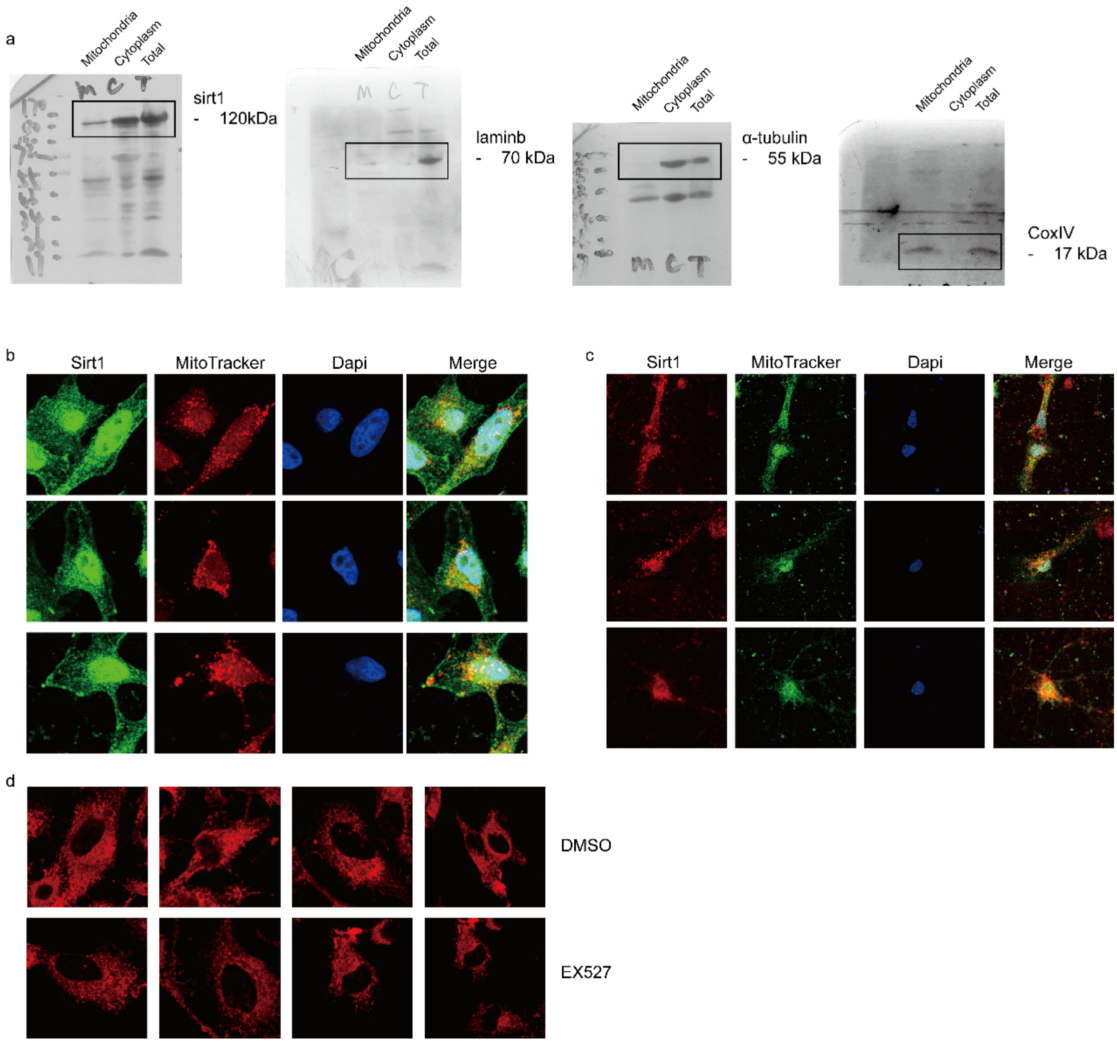

Supplementary Figure S1.

a. Full blots of Western blot analysis of SIRT1 expression in mitochondria, cytoplasm and whole-cell lysates of Hela cells.

b. Additional images of the colocalization of SIRT1 and MitoTracker in Hela cells.

c. Additional images showing the colocalization of SIRT1 and MitoTracker in rat cortical neurons.

d. Additional images showing the morphology of Hela cells treated with EX527.

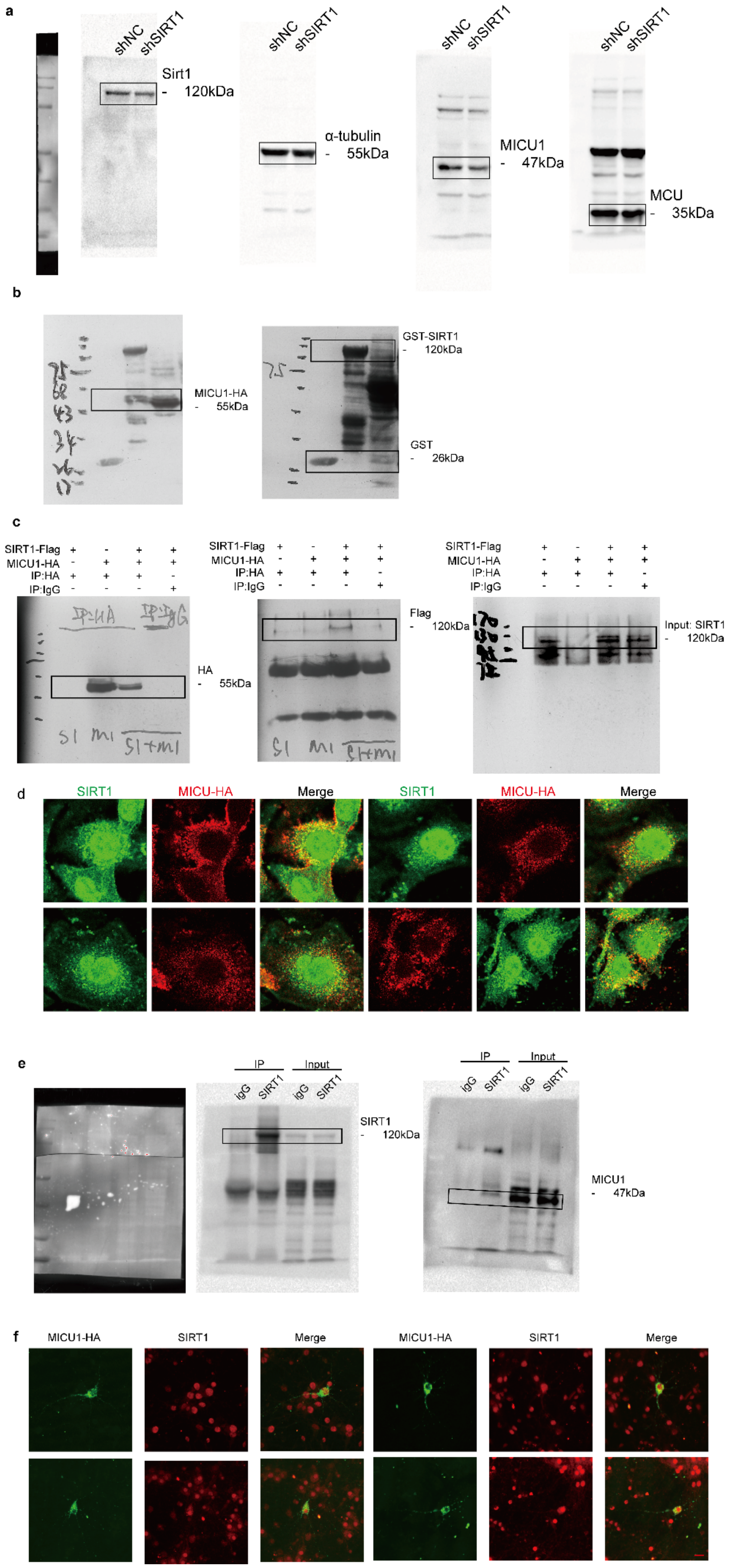
a
shNC
shSIRT1
Sirt1
- 120kDa
α-tubulin
- 55kDa
MICU1
- 47kDa
MCU
- 35kDa
b
MICU1-HA
- 55kDa
GST-SIRT1
- 120kDa
GST
- 26kDa
c
SIRT1-Flag
MICU1-HA
IP:HA
IP:IgG
HA
- 55kDa
Flag
- 120kDa
Input: SIRT1
- 120kDa
d
SIRT1
MICU-HA
Merge
e
IP
Input
igG
SIRT1
SIRT1
- 120kDa
MICU1
- 47kDa
f
MICU1-HA
SIRT1
Merge

Supplementary Figure S2

a. Full blots of Western blot analysis of SIRT1, MCU and MICU1 protein expression levels.
b. Full blots of Western blot analysis of the GST pull down assay.
c. Full blots of Western blot analysis of the co-IP assay in HeLa cells.
d. Additional images of the colocalization of SIRT1 and MICU1-HA.
e. Full blots of Western blot analysis of the co-IP assay in primary cultured rat neurons.
f. Supplementary images of primary cultured rat neurons transfected with MICU1-HA. Red, SIRT1; Green, HA. Magnification is 60X. Scale bars, 50 μm.
